# IgG4-related disease: a case report with duration of more than 16 years and review of literature

**DOI:** 10.1186/s40064-016-2537-2

**Published:** 2016-06-21

**Authors:** Tao Peng, Zhao Hu, Tingting Xie, Baodong Jiang, Junhui Zhen, Xiangdong Yang

**Affiliations:** Department of Nephrology, Shandong University Qilu Hospital, Jinan, 250012 China

**Keywords:** IgG4-related disease, Chronic kidney disease, Prognosis, Pseudotumor, Glucocorticoids, Misdiagnosis

## Abstract

**Introduction:**

IgG4-related disease was first reported in 2001 and was officially named in 2010. It is now considered as a systemic disease that might affect any organ system. The characteristic pathological changes of IgG4-related disease are extensive infiltration of IgG4-positive plasma cells. IgG4-related disease is a kind of benign lesions, but there has not been well-defined standard treatment so far. Patients usually respond well to corticosteroids. The prognosis of IgG4-related disease is perhaps good as long as early detection and treatment.

**Case description:**

We report one case of IgG4-related disease with a 16-years anamnesis with multi-pseudotumor masses. He was diagnosed with chronic kidney disease with wide interstitial renal fibrosis. And he received glucocorticoids therapy. After 2 month therapy, the serum creatinine, erythrocyte sedimentation rate, and serum IgG4 decreased significantly. The discussion includes presentation, clinical course, diagnosis, and prognosis of IgG4-related disease. The case and discussion highlight the importance of diagnosis and the good prognosis of IgG4-related diseases.

**Discussion and evaluation:**

Our case highlights the importance of diagnosis and the good prognosis of IgG4-related diseases. IgG4-related disease is a systemic fibro-inflammatory immune-mediated disorder and now recognized in almost every major organs. Characteristics of the disease is multiple lymph nodes and the response to glucocorticoids therapy is well. In such case, he had a history of 16 years with multi-pseudotumor masses and misdiagnosed for 16 years, if the doctors were not awareness of higher serum immunoglobulin G4 (IgG4) than normal, the correct diagnosis may be missed or delayed. Consequently, appropriate treatment for IgG4-related disease would also be delayed or not provided and likely result in increased morbidity and mortality.

**Conclusions:**

IgG4-related disease is a systemic fibro-inflammatory immune-mediated disorder and progresses slowly. In the present patient the course of IgG4-related disease appears to be benign. The prognosis of IgG4-related disease depend on early diagnosis and treatment.

## Introduction

IgG4-related disease is now considered as a systemic disease that might affect any organ system, including kidneys, lymph nodes, and thyroid gland, with progressively growing fibro-inflammatory lesions causing a mass effect. Diagnostic criteria were established as: IgG4 plasma level of >135 mg/dl and an IgG4/IgG plasma cell ratio of >40 % with >10 and IgG4-positive plasma cells per HPF. Macroscopically, these diseases cause diffuse organ swelling and formation of pseudotumor masses. Patients usually respond well to corticosteroids, but highly active diseases may require other immunosuppressive therapies. IgG4-related disease was first reported in 2001, which came from autoimmune pancreatitis (AIP) and was officially named in 2010. The prognosis of the disease has not been clearly defined. We hereby reported a 54-year-old male with IgG4-related disease, a history of 16 years with multi-pseudotumor masses, he was diagnosed as chronic kidney disease with Scr 545 µmol/L and interstitial renal fibrosis widely. And the response to glucocorticoids therapy was well. After 1 month therapy, the Serum creatinine (Scr), erythrocyte sedimentation rate (ESR), and IgG4 decreased significantly.

## Case report

IgG4-related disease was diagnosed in a 54-year-old male with lumps in both orbital cavity for more than 16 years and lymphadenopathy in mediastinum for 11 years prior to hospital admission. In March 2000, a 54-year-old man was present in a local hospital because of exophthalmos for one year. The physical examination showed that there was hard lumps out of the top of left orbital cavity (0.5 × 0.5 cm) and larger tender bilateral submandibular lymph nodes. A computed tomography (CT) scanning and ophthalmic ultrasound indicated that occupying lesion located in left orbital cavity. Scr was 76 µmol/L. Pathology of lumps in the orbit was “inflammatory pseudotumor (benign lymphoma)”, and he was given prednisone, 30 mg qd. After 2 weeks, bilateral submandibular lymph nodes shrunk significantly (0.3 × 0.3 cm).

During April 2000 to May 2004, the patient did not go to see any doctor for the above disease. At June 2004, he received CT scanning examination, and there still was swelling lymph nodes in mediastinum and abdomen. He was given prednisone (unknown exact dose and duration, but he refused following-up in local hospital. At January 2015, he went to local hospital because of constipation and abdominal pain. Examination results showed that Scr elevated (396 µmol/L) and proteinuria was positive (+), then he accepted some relative treatments. But at early of May 2015, Scr increased to 488 µmol/L, so he was admitted to our hospital. His prior medical history also included mild lower extremity numbness for more than 5 years.

Results of a physical examination revealed that there were enlarged lymph nodes behind the left ear, right groin and left orbital cavity, each was about 1.0 × 1.0 cm. There was no malar rash, oral ulcers, diffuse alopecia or edema in bilateral eyelids and lower limbs. Pertinent laboratory findings included an ESR of 101 mm/h. White blood cell count, platelet count, and hemoglobin were all normal. Parathyroid hormone (PTH) was 351 pg/ml. Ca^2+^ and P^3+^ were normal. Anti-glomerular basement membrane antibody and anti-neutrophil antibody was negative. The ratio of light chain κ/λ in urine and blood were normal. Urinary albumin-creatinine ratio (ACR) was 0.25 g/gCr. The serum immunoglobulin G (IgG) was 57,500 mg/L (reference range 20,000–40,000 mg/L) accounting for 50.3 % of total immunoglobulin, significantly higher than normal (normal 9–16 %). Serum immunofixation electrophoresis was negative. Level of C3 and C4 were normal.

After admission, laboratory tests were positive for serum immunoglobulin G4 (IgG4), 38,500 mg/L (normal 30–2010 mg/L), accounting for 66.9 % of total IgG. CT scanning showed there were enlarged lymph nodes in the left side of face, in bilateral orbital cavity, and bilateral temporal fossa. Scanning of CT indicated that there were thyroid nodules and enlarged lymph nodes in mediastinum, abdominal cavity, and retroperitoneum. The morphology of peripheral blood cell was normal.

**Fig. 1:**
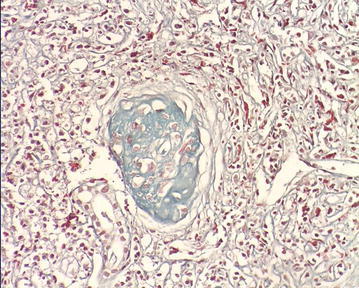
A large number of plasma cell diffuse infiltration and IgG4 staining was positive and tubules atrophy, renal interstitial diffuse fibrosis. IgG4-immunostaining and Masson staining (×400)

**Fig. 2:**
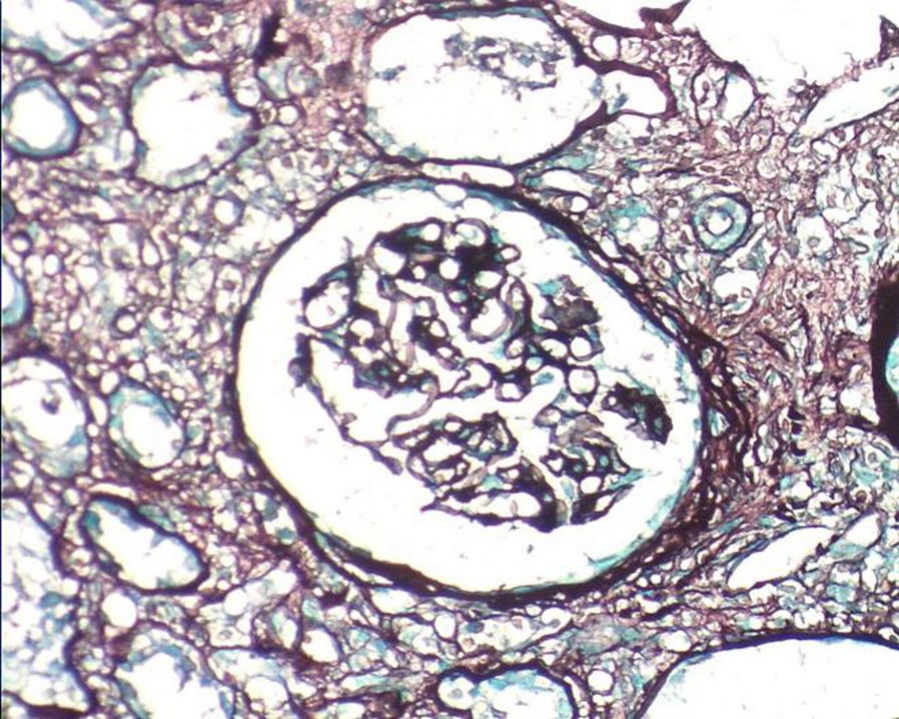
A large number of plasma cell diffuse infiltration and IgG4 staining was positive and tubules atrophy, renal interstitial diffuse fibrosis. PASM (Periodic acid-silver metheramine) staining (×400)

**Fig. 3:**
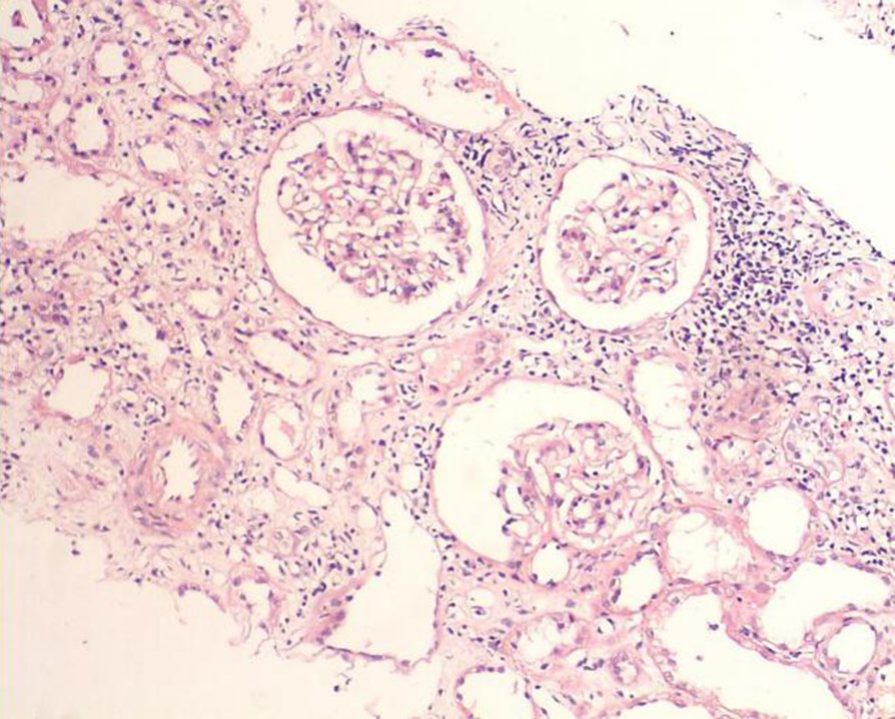
A large number of plasma cell diffuse infiltration and IgG4 staining was positive and tubules atrophy, renal interstitial diffuse fibrosis. HE (Hematoxylin-eosin) staining (×200)

The patient accepted renal biopsy but refused lymph node biopsy. We obtained two renal biopsy specimens, and each specimen with length of about 1 cm contained 100 % cortex with 13 glomeruli. On light microscopy of hematoxylin-eosin staining, there was total 13 glomeruli, including 6 glomeruli completely fibrosis and 3 glomeruli peribulbar fibrosis. The mesangial cell and matrix showed no proliferation, there was no thickening of glomerular capillary. In Masson staining, no immune complexes deposited in capillary wall. In PASM staining, 60 % of tubules were atrophic, others showed dilatation with flat epithelial cells, renal interstitial showed diffuse fibrosis with a large number of plasma cell diffuse infiltration and IgG4 staining was positive (average 47 IgG4-positive plasma cells per high power field) (Figs. 1, 2,3).

The result of electron microscopy showed: there was no significant proliferation and expansion in mesangial cells and matrix. The capillary wall basement membrane showed diffuse thickening and shrinkage, without significant electron dense deposit. Foot process of podocyte appeared diffuse effacement. Tubular epithelial cell and organelle swollen. Tubular basement membrane was almost normal. The interstitium showed focal edema, fibrosis with inflammatory cell infiltration.

According to clinical manifestations, laboratory findings, and imaging results, the case was diagnosed as IgG4-related disease. He was managed with standard prednisolone with relative drugs. Ten days later, Scr and ESR decreased. Enlarged lymph nodes behind of left ear, right groin and left orbital cavity, became significantly smaller 10 days after starting steroid therapy, each was about 0.5 × 0.5 cm. Lower extremity numbness also mitigated.

One month later, he returned for re-examination. Enlarged lymph nodes in the behind of left ear, right groin and left orbital cavity disappeared. Lower extremity numbness also disappeared. ESR decreased to 15 mm/h, Scr decreased to 311 µmol/L, urinary ACR was 0.2 g/gCr and level of IgG4 decreased to 8500 mg/L. Enlarged lymph nodes became smaller than before in CT. Two months later, level of IgG4 decreased to 2060 mg/L.

## Discussions

IgG4-related disease is a disease associated with serum increased IgG4, which is involved in chronic and autoimmune disease in multiple organs and/or tissues (Grados et al. 2015), including many kinds of diseases (Furukawa et al. 2015; Yakirevich et al. 2015; Nishikawa et al. 2014; Kragstrup et al. 2013; Ahn et al. 2014; Huggett et al. 2014; Pusztaszeri et al. 2012; Hori et al. 2010), for example, inflammatory pseudotumor (Choi et al. 2015), Mikulicz`s disease (Takano et al. 2014), and autoimmune pancreatitis (AIP) (Yakirevich et al. 2015). AIP often expresses acute or chronic pancreatitis and imaging scanning shows characteristic sausage-type swelling of the pancreas (Hara et al. 2014; Brito-Zerón et al. 2014; Eu Jin et al. 2010). The relationship of AIP and IgG4-positive plasma cells was introduced and presented for the first time in 2001 with a typical biopsy specimen revealed a large number of IgG4-positive plasma cell infiltration. IgG4 systemic disease was also called IgG4-related disease or lgG4-related multi-organ lymphoproliferative syndrome (Pieringer et al. 2014). In the year 2010, IgG4-related disease were announced to birth on Autoimmun Rev magazine (Yoshida et al. 1995; Takahashi et al. 2010). In the present report, the patient was diagnosed inflammatory pseudotumor at first time in the year 2000, and after 15 years, he was finally diagnosed as IgG4-related disease.

The characteristic pathological changes of IgG4-related disease are extensive infiltration of IgG4-positive plasma cells in tissues and multiple organs, which lead to fibrosis (Martinez et al. 2014). CD4^+^ and CD8^+^ T cells may be involved in occurrence of IgG4-related disease. In addition, long-term asbestos exposure may be a predisposing factor (Toyoshima et al. 2010). There are some common characteristics in IgG4-related disease, including swelling and sclerosis in one or more organs or tissues, similar to tumor; IgG4/IgG plasma cell ratio >40 % with >10 and IgG4-positive plasma cells per HPF; IgG4 plasma level >1350 mg/L; good response to glucocorticoid therapy.

In present study, the patient had lumps in both orbital cavity for 16 years, swelling lymph nodes in mediastinum and abdomen at least for 11 years and the pathology of lumps in the orbit was diagnosed as “inflammatory pseudotumor (benign lymphoma)” in the year 2000. Because of the limited medical knowledge of IG4-related systemic disease in the year 2000, IgG4 staining did not completed at that time. Unfortunately the specimens at diagnosis were lost and we could not perform IgG4 staining. CT scanning at different timepoints showed enlarged lymph nodes in many organs. Laboratory tests was positive, 38,500 mg/L, for IgG4, >1350 mg/L, accounting for 66.9 % of total IgG (>40 %). In renal biopsy, 60 % tubules was atrophy, other tubules showed dilatation with flat epithelial cells, renal interstitial showed diffuse fibrosis with diffuse infiltration of a large number of plasma cell and IgG4 staining was positive (Figs. 1, 2,3) (Cortazar and Stone 2015).

 Previous reports showed that IgG4-related disease is a kind of benign lesions, but there has not been well-defined standard treatment so far. It was generally sensitive to glucocorticoids (Wallace and Stone 2015; Hasegawa et al. 2015). After glucocorticoids treatment, organs or tissues swelling subsided significantly, serum IgG4 decreased and clinical manifestations were ameliorated significantly, but if discontinuing glucocorticoid, it may relapses. In the present study, the patient received prednisone, 30 mg qd, for 2 weeks in the year 2000. After 14 days, lymph nodes in bilateral submandibular became significantly smaller. And in the year 2015, he received again prednisolone. At 10-day-, 1-month- and 2-month following-up, Scr, IgG4, and ESR significantly decreased. Enlarged lymph nodes became significantly smaller.

It has been only 15 years since the first IgG4-related system diseases was reported Takahashi et al. (2010), and the long-term prognosis of IgG4-related systemic was still unclear. In the present study it was more than 16 years since the lumps in both orbital cavity first found.During this period, he did not receive systemic therapy. Because of increased Scr, he went to see a doctor and IgG4-related disease was diagnosed finally. The result indicated that IgG4-related disease could be a kind of benign lesions, and progress very slowly even without treatment.

## Conclusions

IgG4-related disease is a systemic fibro-inflammatory immune-mediated disorder and now recognized in many organs. It is characterised with multiple enlarged lymph nodes and well response to glucocorticoids. IgG4-related disease progresses slowly,and in the present patient the course of IgG4-related disease appears to be benign. The prognosis of IgG4-related disease is perhaps good as long as early detection and treatment.

## References

[CR1] Ahn JH, Hong SI, Cho DH, Chae EJ, Song JS, Song JW (2014). A case of IgG4-related lung disease presenting as interstitial lung disease. Tuberc Respir Dis (Seoul).

[CR2] Brito-Zerón P, Ramos-Casals M, Bosch X, Stone JH (2014). The clinical spectrum of IgG4-related disease. Autoimmun Rev.

[CR3] Choi YJ, Lee MJ, Kim N, Choung HK, Khwarg SI, Kim JE (2015). Inflammatory pseudotumor of eyelid: a probable IgG4-related sclerosing disease clinically mimicking eyelid pilomatrixoma. BMC Ophthalmol.

[CR4] Cortazar FB, Stone JH (2015). IgG4-related disease and the kidney. Nat Rev Nephrol.

[CR6] Eu Jin L, Bhathal PS, Tagkalidis PP, Speer AG (2010). Catching a chameleon: IgG4-related disease. Med J Australia.

[CR7] Furukawa S, Moriyama M, Tanaka A, Maehara T, Tsuboi H, Iizuka M, Hayashida JN, Ohta M, Saeki T, Notohara K, Sumida T, Nakamura S (2015). Preferential M2 macrophages contribute to fibrosis in IgG4-related dacryoadenitis and sialoadenitis, so-called Mikulicz’s disease. Clin Immunol.

[CR8] Grados A, Ebbo M, Jean E, Bernit E, Harlé JR, Schleinitz N (2015). IgG4-related disease treatment in 2014: update and literature review. Rev Med Intern.

[CR10] Hara N, Kawaguchi M, Takeda K, Zen Y (2014). Retroperitoneal disorders associated with IgG4-related autoimmune pancreatitis. World J Gastroenterol.

[CR11] Hasegawa S, Mine S, Hagiwara S (2015). IgG4-related disease combined with autoimmune hemolytic anemia and steroid-responsive transient hypercalcemia. Clin Med Insights Case Rep.

[CR12] Hori M, Makita N, Andoh T, Takiyama H, Yajima Y, Sakatani T, Fukumoto S, Iiri T, Fujita T (2010). Long-term clinical course of IgG4-related systemic disease accompanied by hypophysitis. Endocr J.

[CR13] Huggett MT, Culver EL, Kumar M, Hurst JM, Rodriguez-Justo M, Chapman MH, Johnson GJ, Pereira SP, Chapman RW, Webster GJ, Barnes E (2014). Type 1 autoimmune pancreatitis and IgG4-related sclerosing cholangitis is associated with extrapancreatic organ failure, malignancy, and mortality in a prospective UK cohort. Am J Gastroenterol.

[CR14] Kragstrup TW, Vorup-Jensen T, Deleuran B, Hvid MA (2013). Simple set of validation steps identifies and removes false results in a sandwich enzyme-linked immunosorbent assay caused by anti-animal IgG antibodies in plasma from arthritis patients. Springerplus.

[CR15] Martinez LL, Friedländer E, van der Laak JA, Hebeda KM (2014). Abundance of IgG4+ plasma cells in isolated reactive lymphadenopathy is no indication of IgG4-related disease. Am J Clin Pathol.

[CR17] Nishikawa K, Takeda A, Masui S, Kanda H, Yamada Y, Arima K, Morozumi K, Sugimura Y (2014). A case of IgG4-positive plasma cell-rich tubulointerstitial nephritis in a kidney allograft mimickingIgG4-related kidney disease. Nephrology (Carlton).

[CR19] Pieringer H, Parzer I, Whrer A, Reis P, Oppl B, Zwerina J (2014). IgG4- related disease: an orphan disease with many faces. Orphanet J Rare Dis.

[CR20] Pusztaszeri M, Triponez F, Pache JC, Bongiovanni M (2012). Riedel’s thyroiditis with increased IgG4 plasma cells: evidence for an underlying IgG4-related sclerosing disease?. Thyroid.

[CR21] Takahashi H, Yamamoto M, Suzuki C, Naishiro Y, Shinomura Y, Imai K (2010). The birthday of a new syndrome: IgG4-related diseases constitute a clinical entity. Autoimmun Rev.

[CR22] Takano K, Yajima R, Seki N, Abe A, Yamamoto M, Takahashi H, Himi T (2014). A study of infraorbital nerve swelling associated with immunoglobulin G4 Mikulicz’s disease. Mod Rheumatol.

[CR24] Toyoshima M, Chida K, Kono M, Kaida Y, Nakamura Y, Suda T, Sugimura H (2010). IgG4-related lung disease in a worker occupationally exposed to asbestos. Intern Med.

[CR25] Wallace ZS, Stone JH (2015). An update on IgG4-related disease. Curr Opin Rheumatol.

[CR26] Yakirevich E, Henriksen KJ, Miner T, Resnick MB (2015). Mucinous cystic neoplasm of the pancreas with increased IgG4+ plasma cells and histopathologic features of autoimmune pancreatitis/IgG4-related disease. Pancreas.

[CR27] Yoshida K, Toki F, Takeuchi T, Watanabe S, Shiratori K, Hayashi N (1995). Chronic pancreatitis caused by an autoimmune abnormality. Proposal of the concept of autoimmune pancreatitis. Dig Dis Sci.

